# Mini‐Mental State Examination trajectories across dementia subtypes in the Swedish Registry for Cognitive/Dementia Disorders and U.S. National Alzheimer's Coordinating Center

**DOI:** 10.1002/dad2.70531

**Published:** 2026-10-30

**Authors:** Xinqi Bao, Belén García Pascual, Eirik Blakstad, Jakob Norgren, Tamar Abzhandadze, Nanbo Zhu, Minh Tuan Hoang, Saikat Chatterjee, Martina Scolamiero, Maria Eriksdotter, Hong Xu, Sara Garcia Ptacek

**Affiliations:** ^1^ Department of Neurobiology, Care Sciences and Society Karolinska Institutet Stockholm Sweden; ^2^ Department of Information Science and Engineering School of Electrical Engineering and Computer Science KTH Royal Institute of Technology Stockholm Sweden; ^3^ Department of Mathematics KTH Royal Institute of Technology Stockholm Sweden; ^4^ Department of Occupational Therapy and Physiotherapy Sahlgrenska University Hospital Mölndal Sweden; ^5^ Faculty of Public Health University of Medicine and Pharmacy Vietnam National University Hanoi Vietnam; ^6^ Aging and Inflammation Theme Karolinska University Hospital Stockholm Sweden; ^7^ Department of Women's and Children's Health Division of Clinical Paediatrics Karolinska Institutet Stockholm Sweden

**Keywords:** cognitive decline, cross‐cohort comparison, longitudinal trajectories, mixed‐effects models, real‐world data

## Abstract

**INTRODUCTION:**

Mini‐Mental State Examination (MMSE) trajectories are widely used to describe cognitive decline in dementia, but estimates may be sensitive to cohort structure, follow‐up density, and modeling assumptions. We compared subtype‐specific MMSE trajectories across two complementary longitudinal data resources.

**METHODS:**

The analyzed data were from the Swedish Registry for Cognitive/Dementia Disorders (SveDem) and the U.S. National Alzheimer's Coordinating Center (NACC). Participants were included with at least two valid cognitive assessments separated by ≥ 90 days. The final cohorts included 30,115 SveDem participants and 33,429 NACC participants. Subtype‐stratified regression‐spline generalized additive mixed models and linear mixed‐effects models were fitted with adjustment for demographic and clinical covariates.

**RESULTS:**

Compared with NACC, SveDem participants were older (median age 79 vs. 72 years), had lower baseline MMSE scores (22.3 ± 4.2 vs. 26.5 ± 4.6), and had shorter follow‐up (median 1.9 vs. 3.2 years; mean cognitive assessments 2.9 vs. 4.5). Shared dementia subtypes showed broadly similar relative decline patterns across cohorts. In regression‐spline models, Alzheimer's disease showed faster estimated annualized decline than vascular dementia in both SveDem (−0.79 vs. −0.49 MMSE points/year) and NACC (−1.22 vs. −0.24 points/year). Lewy body dementias and frontotemporal dementia showed intermediate but less consistent patterns. Absolute decline estimates varied by model specification, particularly where later follow‐up was sparse.

**DISCUSSION:**

Relative subtype‐specific MMSE decline patterns were broadly reproducible, whereas absolute long‐horizon estimates required cautious interpretation. These cross‐cohort reference patterns may support future dementia epidemiology studies using MMSE trajectories to compare subtypes, examine clinical factors or evaluate treatment‐related associations.

## Highlights

- Alzheimer's disease showed faster Mini‐Mental State Examination (MMSE) decline than vascular dementia in two large longitudinal dementia cohorts.
- Relative subtype‐specific decline patterns were more stable across cohorts than absolute annualized decline estimates.
- Cross‐cohort MMSE trajectory patterns provide reference estimates for future observational studies and interventional trials.

## 1 BACKGROUND

Dementia comprises heterogeneous neurodegenerative and vascular disorders that differ in clinical phenotype and pace of cognitive deterioration. Major etiologic subtypes include Alzheimer's disease (AD), vascular dementia (VaD), Lewy body dementias (LBD), and frontotemporal dementia (FTD), which may show different longitudinal patterns of decline. Such information is relevant to prognosis, care planning, and the design of studies that use cognitive change as an outcome.^1^, ^2^

The Mini‐Mental State Examination (MMSE) remains one of the most widely used measures of global cognition in routine care, observational research, and clinical trials.^3^, ^4^, ^5^ However, MMSE is a brief and bounded scale. Observed change may reflect not only disease progression, but also ceiling and floor effects, variable sensitivity across disease stages, measurement variability, and differences in follow‐up implementation.^4^, ^5^ These features make the analysis and interpretation of subtle longitudinal differences challenging.

Most longitudinal studies of cognitive decline rely on a single cohort. This makes it difficult to determine whether observed subtype‐specific patterns represent reproducible disease‐related differences or features of a particular data source, recruitment setting or follow‐up structure. The Swedish Registry for Cognitive/Dementia Disorders (SveDem) and the U.S. National Alzheimer's Coordinating Center (NACC) provide a useful contrast. SveDem reflects routine clinical dementia care in Sweden, whereas NACC provides standardized research follow‐up across U.S. Alzheimer's Disease Research Centers.^6^, ^7^, ^8^ Differences in case‐mix, diagnostic workflow and assessment density between these resources create an opportunity to examine which MMSE‐based trajectory findings are stable across settings and which are dependent on cohort structure.

Longitudinal MMSE analysis is further complicated by irregular visit spacing, sparse repeated measurements, informative attrition and possible departures from constant linear change.^9^, ^10^ Linear mixed‐effects models are widely used, but assume a constant average rate of change over time. Flexible approaches such as regression‐spline generalized additive mixed models allow the mean trajectory to vary over follow‐up while retaining participant‐level random effects.^11^ Comparing linear and flexible time specifications can therefore help assess how strongly long‐horizon decline estimates depend on modeling assumptions.

This study compared subtype‐specific MMSE trajectories across SveDem and NACC. The primary aim was to determine whether relative patterns of cognitive decline across dementia subtypes were reproducible across two complementary longitudinal data resources. A secondary aim was to examine whether absolute long‐horizon decline estimates differed according to cohort follow‐up structure and time‐model specification. The study was intended to provide cross‐cohort reference patterns for future studies using MMSE trajectories to compare dementia subtypes, examine clinical factors or evaluate treatment‐related associations.

## 2 METHODS

### 2.1 Data sources and study population

This study analyzed longitudinal cognitive data from SveDem and NACC. SveDem is a nationwide Swedish clinical quality registry recording demographic characteristics, clinician‐reported dementia diagnoses and repeated cognitive assessments, mainly MMSE, from routine care.^6^ SveDem comorbidity indicators were derived from International Classification of Diseases (ICD) ‐based diagnoses in the Swedish National Patient Register.^12^, ^13^, ^14^ NACC is a standardized longitudinal research dataset from U.S. Alzheimer's Disease Research Centers, including repeated clinical, diagnostic and neuropsychological assessments.^7^, ^8^ Medical conditions in NACC are recorded through participant or informant report with clinician documentation.^8^ Global cognitive assessments include MMSE and the Montreal Cognitive Assessment (MoCA).^8^, ^15^

Participant selection is summarized in Figure 1, with demographic and diagnostic distributions after each filtering step provided in Figure S1. In SveDem, 107,307 individuals were initially available; after excluding missing key baseline variables, 73,700 remained for longitudinal screening, and 30,115 met the criteria of at least two valid cognitive assessments separated by ≥ 90 days. In NACC, 54,631 individuals were available at baseline; 51,554 remained after analogous baseline completeness criteria, and 33,429 met the longitudinal follow‐up criteria.

**RESEARCH IN CONTEXT**

1. **Systematic review**: Previous studies of cognitive decline across dementia sub‐types have largely used single cohorts or specialist clinical samples. The Mini‐Mental State Examination (MMSE) remains widely used for longitudinal disease monitoring, but differences in case‐mix, follow‐up structure, diagnostic definitions, and modeling approaches complicate comparisons across datasets. Direct cross‐cohort evaluations of subtype‐specific MMSE trajectories remain limited.
2. **Interpretation**: Using two large and complementary dementia data resources, this study found broadly comparable relative MMSE decline patterns across Swedish Registry for Cognitive/Dementia Disorders (SveDem) and U.S. National Alzheimer's Coordinating Center (NACC), particularly faster decline in Alzheimer's disease than in vascular dementia. In contrast, absolute long‐horizon decline estimates varied across cohorts and time‐model specifications, especially where later follow‐up was sparse. Relative subtype patterns may therefore be more transportable across cohorts than absolute annualized decline estimates.
3. **Future directions**: Future longitudinal studies should report the observational support at later follow‐up and explicitly describe diagnostic definitions, cognitive‐score harmonization, attrition, and time‐model assumptions. Replication across routine‐care registries and research cohorts is needed to determine whether MMSE‐based associations with clinical factors or treatments are robust to differences in population selection and data structure

**FIGURE 1:**
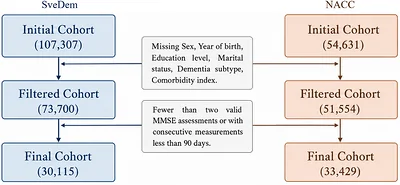
Participant‐selection flow in SveDem and NACC. Individuals were excluded for missing baseline demographic and diagnostic information and then for having fewer than two valid cognitive assessments or intervals shorter than 90 days between consecutive assessments. NACC, U.S. National Alzheimer's Coordinating Center; SveDEM, Swedish Registry for Cognitive/Dementia Disorders.

### 2.2 Dementia subtype classification

Dementia subtype definitions were harmonized to support cross‐database comparison while preserving cohort‐specific diagnostic structures. The primary dementia subtypes were AD, VaD, LBD (including dementia with Lewy bodies and Parkinson's disease dementia where available), and FTD. Mixed dementia, defined as overlapping AD and VaD diagnoses, was modeled in SveDem only because no directly comparable NACC category was available. Cognitively unimpaired participants and individuals with mild cognitive impairment were additionally modeled as reference groups in NACC. Dementia‐specific trajectory models were restricted to the specified dementia subtypes.

In SveDem, subtype was defined at registry entry, with initially unspecified cases reassigned if a more specific diagnosis was recorded during follow‐up.^6^ This classification was used to describe subsequent MMSE trajectories following entry into clinical follow‐up. In NACC, cognitive status and dementia etiology were updated at each visit,^7^, ^8^ and each retained visit was assigned to the state recorded at that visit. Participants whose recorded state changed, therefore, could contribute visits to different state‐specific models, while follow‐up time remained anchored to the first eligible cognitive assessment and was not reset at the transition.

### 2.3 Outcome and follow‐up definition

The primary outcome was global cognitive performance measured using the MMSE, which ranges from 0 to 30, with lower scores indicating worse cognition.^3^ In NACC, MoCA scores were converted to MMSE‐equivalent values using a validated calibration approach to maximize longitudinal coverage.^15^, ^16^ Primary NACC analyzes therefore combined observed MMSE and MoCA‐equated MMSE values, with MMSE‐only analyzes performed as sensitivity analyzes. This harmonization was not applied in SveDem because repeated MoCA assessments were rare and basically overlapped with MMSE data.

Baseline MMSE was defined at the first retained cognitive assessment and mean‐centered before model fitting. Follow‐up time was calculated in years from this assessment. To reduce short‐term retesting effects, subsequent assessments were retained only if they occurred at least 90 days after the previous retained assessment. Participants contributed observations until their last available cognitive assessment.

### 2.4 Covariates and harmonization

Baseline covariates were harmonized across cohorts and included age, sex, years of education, marital status, shared comorbidity count, and baseline MMSE. Age was *cal*culated from year of birth and the year of the first retained cognitive assessment. Sex was harmonized to a binary indicator. SveDem was linked to the Longitudinal Integrated Database for Health Insurance and Labour Market Studies to obtain education and civil‐status information. Education was harmonized to years of schooling using cohort‐specific mappings, and marital status was recoded as married/cohabiting, widowed, or single/divorced.^17^, ^18^ Detailed harmonization rules are provided in the Supplementary Methods and Table S1.

The shared comorbidity count was an unweighted sum of 11 chronic disease indicators available in both cohorts and was used as a pragmatic measure of recorded comorbidity rather than a full Charlson score.^12^, ^19^ Details are provided in the Supplementary Methods. No imputation was applied after cohort construction.

### 2.5 Statistical modeling

Analyzes were performed on long‐format data and stratified by database and dementia subtype. For each subtype g, a regression‐spline generalized additive mixed model (GAMM) was fitted with a flexible time function and a subject‐specific random intercept:

$$Y_{ijg}=\beta_{0g}+f_{g}\left(t_{ij}\right)+\gamma_{g}^{⊤}X_{i}+b_{ig}+\varepsilon_{ijg}$$

where $Y_{\mathrm{ijg}}$ is MMSE (or MoCA‐equated MMSE in NACC) for individual *i* at visit *j*; $t_{\mathrm{ij}}$ is time since the first valid cognitive assessment; $X_{i}$ is the vector of baseline covariates; $b_{ig}∼N\left(0,\tau_{g}^{2}\right)$ is a random intercept; and $\varepsilon_{ijg}∼N\left(0,\sigma_{g}^{2}\right)$ is residual error.

The time function $f_{g}\left(\cdot \right)$ was represented using a natural cubic regression spline basis with degrees of freedom capped at 7.^11^ During model development, specifications with 3, 5, and 7 degrees of freedom were examined. The 7‐degree‐of‐freedom specification was retained because it provided sufficient flexibility while preserving smooth, clinically plausible population‐level trajectories in sparsely observed intervals. Random slopes were not included because most individuals contributed only two to three cognitive assessments and random‐slope specifications frequently exhibited unstable estimation or non‐convergence.

As a parametric benchmark, a linear mixed‐effects model (LMM) with the same covariates and random‐intercept structure was fitted, with time modelled linearly:

$$Y_{ijg}=\beta_{0g}+\beta_{1g}t_{ij}+\gamma_{g}^{⊤}X_{i}+b_{ig}+\varepsilon_{ijg}$$

Subtype‐specific mean trajectories were obtained from the fixed‐effect component by evaluating predicted means across a grid of time points spanning the observed follow‐up range and displayed on a common 0‐ to 5‐year window for comparability. Pointwise 95% confidence intervals for predicted means were derived from the fixed‐effect covariance matrix. Because baseline MMSE was included as an adjustment covariate, the adjusted trajectories are conditional on the model‐specific reference covariate profile (Table S2). Additional implementation and model‐fit summaries are provided in Supplementary Methods.

### 2.6 Model comparison

For each database and dementia subtype, the regression‐spline GAMM was compared with the LMM using the same analytic sample, identical baseline covariates, and the same random‐intercept structure.

To summarize the magnitude of change, the model‐based average annual change over 0–5 years for subtype g under model M was defined as

$$\Delta_{g}\left(M\right)=\frac{\mu_{g}^{\left(M\right)}\left(5\right)-\mu_{g}^{\left(M\right)}\left(0\right)}{5},$$

The annualized change was calculated as the difference between the fixed‐effect predicted mean MMSE at year 5 and baseline, divided by 5, at the model‐specific reference covariate profile (Table S2). The corresponding average annual change over 0–3 years was calculated as a sensitivity analysis. Confidence intervals were obtained using the delta method from the fixed‐effect covariance matrix. Negative values indicate decline.

## 3 RESULTS

### 3.1 Cohort characteristics and follow‐up

A total of 30,115 participants from SveDem and 33,429 from NACC met the longitudinal inclusion criteria (Table 1). Participants in SveDem were older than those in NACC (median [interquartile range {IQR}], 79 [73–84] vs. 72 [66–79] years), whereas the proportion of women was similar (57.7% vs. 57.1%). Educational attainment, marital status, subtype composition, and recorded comorbidity profiles differed between the two databases, consistent with their distinct recruitment and data‐capture contexts. SveDem consisted predominantly of dementia cases, whereas NACC included a large proportion of cognitively unimpaired participants and individuals with mild cognitive impairment. Baseline MMSE was lower in SveDem than in NACC (22.3 ± 4.2 vs. 26.5 ± 4.6), and follow‐up was shorter and less dense in SveDem than in NACC (median follow‐up time 1.9 vs. 3.2 years; mean number of cognitive assessments 2.9 vs. 4.5).

**TABLE 1:** Baseline characteristics of the SveDem and NACC cohorts.

Variable	SveDem (*N =* 30,115)	NACC (*N =* 33,429)
**Demographic variables**		
Age, median [IQR]	79 [73–84]	72 [66–79]
Female, n (%)	17,372 (57.7)	19,100 (57.1)

Education level, n (%)		
Primary / below	12,238 (40.6)	1,198 (3.6)
Secondary	14,271 (47.4)	12,159 (36.4)
University / above	3606 (12.0)	20,072 (60.0)

Marital status, n (%)		
Married / cohabiting	14,828 (49.2)	22,249 (66.6)
Widowed	8817 (29.3)	5,158 (15.4)
Single / divorced	6470 (21.5)	6,022 (18.0)
Living alone, *n* (%)	11,667 (38.7)	8,020 (24.0)

Clinical variables		
Comorbidity count, mean ± SD	1.2 ± 1.3	2.1 ± 1.3
Hypertension, *n* (%)	11,504 (38.2)	23,855 (71.4)
Hypercholesterolemia, *n* (%)	3358 (11.2)	23,284 (69.7)
Cardiac ischemic disease, *n* (%)	4160 (13.8)	5,929 (17.7)
Diabetes, *n* (%)	3460 (11.6)	5,653 (16.9)
Cancer (nonmetastatic), *n* (%)	5334 (17.7)	5,129 (15.3)
Stroke / TIA, *n* (%)	4165 (13.8)	3,938 (11.8)
Heart failure, *n* (%)	1901 (6.4)	2,156 (6.5)
Rheumatoid / connective tissue disease, *n* (%)	1362 (4.6)	704 (2.1)
Seizures / epilepsy, *n* (%)	485 (1.6)	523 (1.6)
Metastatic cancer, *n* (%)	275 (0.9)	499 (1.5)

Cognitive state / dementia subtype, *n* (%)		
AD	12,535 (41.6)	6,567 (19.6)
Mixed dementia	5,458 (18.1)	—
VaD	3915 (13.0)	139 (0.4)
FTD	375 (1.2)	970 (2.9)
LBD	990 (3.3)	525 (1.6)
MCI	88 (0.3)	7,632 (22.8)
Unspecified	6754 (22.4)	1,933 (5.8)
Cognitively unimpaired	—	15,663 (46.9)
MMSE at baseline, mean ± SD	22.3 ± 4.2	26.5 ± 4.6
No. of cognitive assessments, mean ± SD	2.9 ± 1.4	4.5 ± 3.0
Follow‐up time, median [IQR], years	1.9 [1.0–3.4]	3.2 [1.6–6.2]

*Not*e: Values are shown as median [IQR], mean ± SD, or *n* (%), as appropriate. Baseline characteristics are presented descriptively.

Abbreviations: AD, Alzheimer's disease; FTD, frontotemporal dementia; IQR, interquartile range; LBD, Lewy body dementias; MCI, mild cognitive impairment; MMSE, Mini‐Mental State Examination; NACC, U.S. National Alzheimer's Coordinating Center; SD, standard deviation; SveDEM, Swedish Registry for Cognitive/Dementia Disorders; TIA, transient ischemic attack; VaD, vascular dementia.

### 3.2 Subtype‐specific trajectories

Figure 2 shows adjusted subtype‐specific MMSE trajectories estimated using regression‐spline GAMM and LMM; unadjusted trajectories are provided in Figure S2. Across both databases, shared dementia subtypes showed broadly similar relative decline patterns. VaD generally showed slower decline and AD showed steeper decline. FTD showed relatively steep decline, particularly in NACC, whereas LBD estimates were more variable across databases. In NACC, cognitively unimpaired participants remained largely stable, and mild cognitive impairment showed modest decline.

**FIGURE 2:**
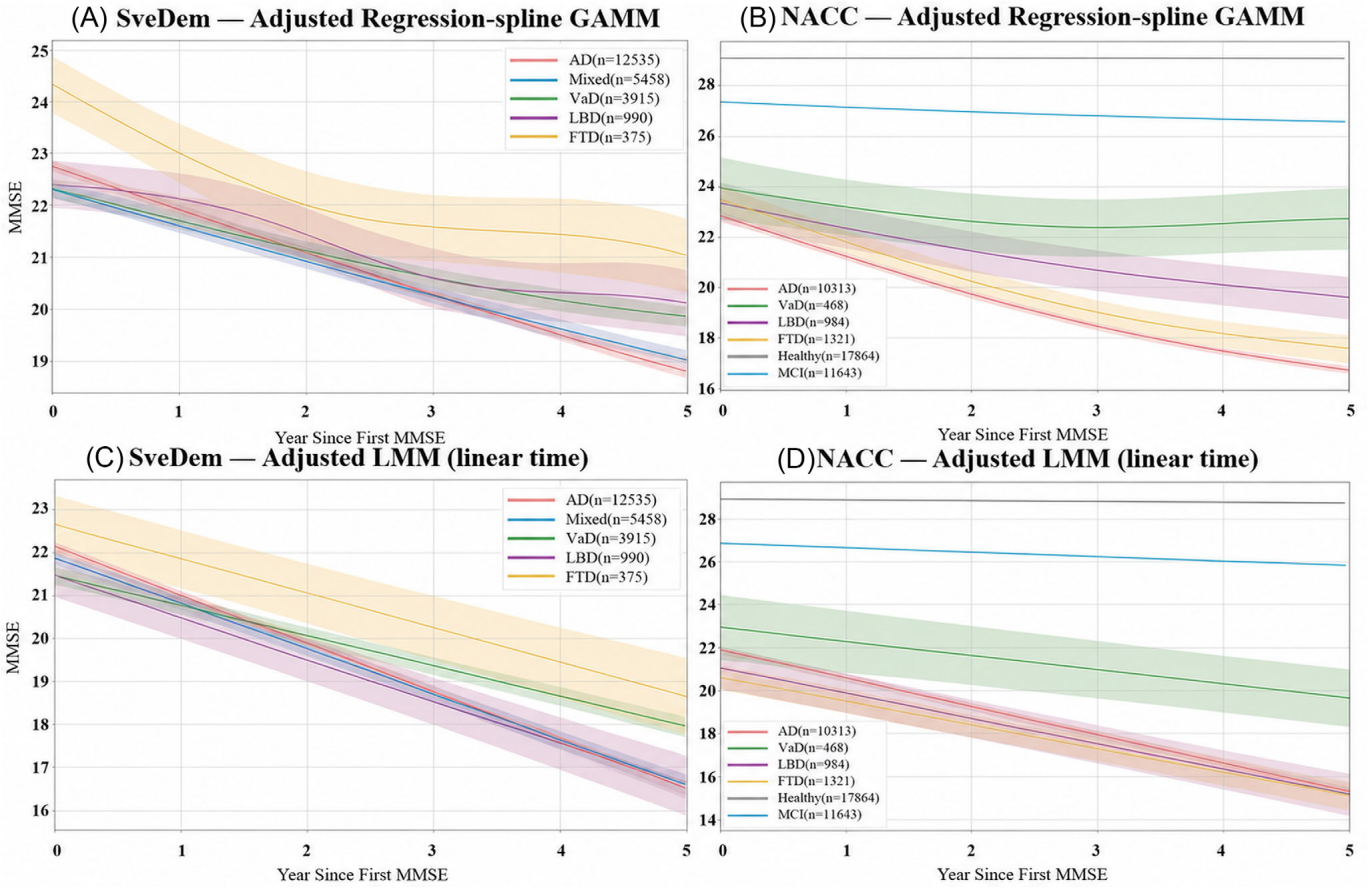
Subtype‐specific MMSE trajectories within each database estimated using adjusted regression‐spline GAMM and adjusted LMM. (A,B) Adjusted regression‐spline GAMM trajectories in SveDem and NACC. (CD) Adjusted LMM trajectories. Curves represent fixed‐effect predictions by years since the first cognitive assessment, and shaded bands indicate 95% confidence intervals. Cognitively unimpaired participants and MCI are shown only for NACC. Numbers in the legends indicate participants recorded in each subtype at one or more retained visits. Because NACC classification was visit‐specific, these categories may overlap across follow‐up and, therefore, differ from the mutually exclusive baseline categories reported in Table 1. Unadjusted trajectories are provided in Figure S2. In the figure, “Healthy” denotes cognitively unimpaired participants. GAMM, generalized additive mixed model; LMM, linear mixed‐effects model; MCI, mild cognitive impairment; MMSE, Mini‐Mental State Examination; NACC, U.S. National Alzheimer's Coordinating Center.

Within each database, LMM and regression‐spline GAMM produced similar patterns during early follow‐up, but diverged more at longer horizons. This was most apparent in SveDem, where later follow‐up was sparser and linear estimates implied steeper long‐horizon decline than spline‐based estimates. In NACC, several subtype trajectories showed modest curvature over longer follow‐up.

### 3.3 Annualized decline and model comparison

Table 2 summarizes adjusted model‐based average annual MMSE change over 0–5 years, together with variance components and explained‐variance summaries, and Figure 3 displays these estimates by subtype, database, and model class. Under the adjusted regression‐spline GAMM, AD showed the steepest estimated decline among the shared dementia subtypes in both databases (SveDem −0.79/year; NACC −1.22/year), whereas VaD showed slower estimated decline (SveDem −0.49/year; NACC −0.24/year). FTD showed relatively steep decline in both databases, particularly in NACC, whereas LBD estimates varied more across databases. Mixed dementia in SveDem declined at −0.65/year.

**TABLE 2:** Adjusted trajectory summaries and variance components for subtype‐specific MMSE models.

Database	Model	Subtype	τ^2^	σ^2^	R^2^ Marginal	R^2^ Conditional	Annualized change
SveDem	Regression‐spline GAMM	AD	2.998	8.063	0.570	0.687	−0.789
		FTD	3.066	10.618	0.543	0.646	−0.661
		LBD	2.690	9.236	0.486	0.602	−0.458
		Mixed dementia	2.154	7.210	0.585	0.680	−0.654
		VaD	2.402	7.736	0.554	0.660	−0.493
	LMM	AD	5.999	8.456	0.566	0.746	−1.136
		FTD	9.281	12.620	0.482	0.702	−0.806
		LBD	4.730	9.628	0.486	0.656	−0.982
		Mixed dementia	4.357	7.504	0.574	0.731	−1.065
		VaD	6.018	8.020	0.533	0.733	−0.707
NACC	Regression‐spline GAMM	AD	6.484	11.334	0.621	0.759	−1.224
		FTD	8.234	17.298	0.606	0.733	−1.194
		Healthy	0.388	1.108	0.375	0.537	−0.011
		LBD	7.682	15.071	0.517	0.680	−0.754
		MCI	1.207	2.986	0.446	0.606	−0.155
		VaD	6.407	10.669	0.536	0.710	−0.244
	LMM	AD	11.489	11.927	0.597	0.795	−1.320
		FTD	14.809	21.729	0.536	0.724	−1.109
		Healthy	10.043	1.124	0.470	0.947	−0.034
		LBD	13.772	16.080	0.498	0.729	−1.183
		MCI	9.050	3.026	0.478	0.869	−0.207
		VaD	17.438	10.799	0.458	0.793	−0.660

*Note*: τ^2^ Denotes random‐intercept variance and σ^2^ denotes residual variance. R^2^ Marginal and R^2^ Conditional denote marginal and conditional explained variance. Annualized change is the model‐based mean annual MMSE change over 0‐5 years, calculated as [μ̂(5) − μ̂(0)]/5; negative values indicate decline.

Abbreviations: AD, Alzheimer's disease; FTD, frontotemporal dementia; GAMM, generalized additive mixed model; LBD, Lewy body dementias; LMM, linear mixed‐effects model; MCI, mild cognitive impairment; NAC, U.S. National Alzheimer's Coordinating Center; MMSE, Mini‐Mental State Examination; VaD, vascular dementia.

**FIGURE 3:**
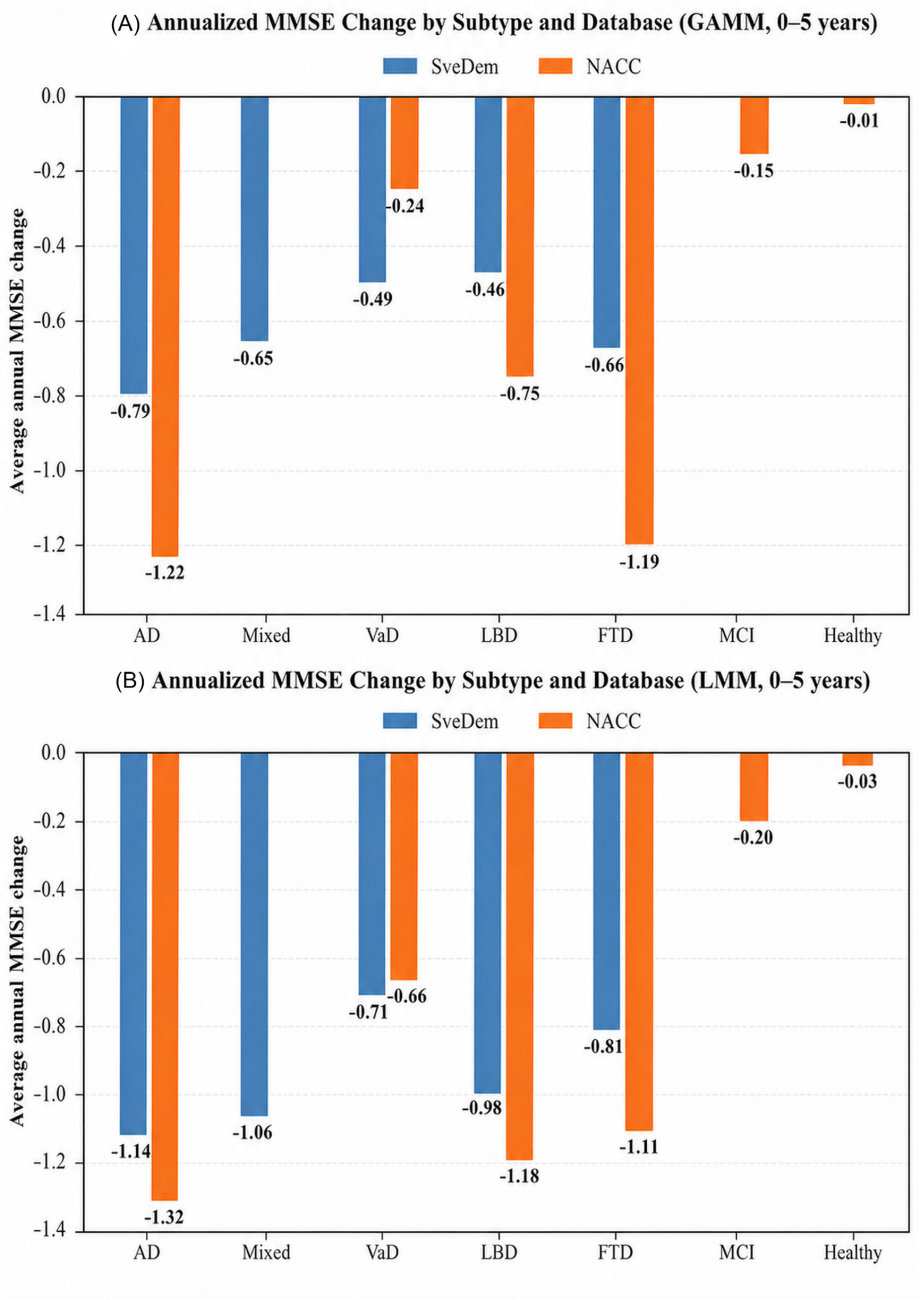
Average annual MMSE change over 0–5 years by subtype and database. (A) Regression‐spline GAMM estimates. (B) Linear LMM estimates. Bars show signed model‐based average annual change (MMSE points/year); negative values indicate decline. Mixed dementia is not defined in NACC and therefore is shown only for SveDem. GAMM, generalized additive mixed model; LMM, linear mixed‐effects model; MMSE, Mini‐Mental State Examination; NACC, U.S. National Alzheimer's Coordinating Center.

The magnitude of estimated decline depended on time‐model specification. In SveDem, the linear‐time LMM generally produced more negative 0‐ to 5‐year average change estimates than the regression‐spline GAMM across subtypes, including AD (−1.14/year vs. −0.79/year), mixed dementia (−1.06/year vs. −0.65/year), LBD (−0.98/year vs. −0.46/year), and VaD (−0.71/year vs −0.49/year). In NACC, differences between the two model classes were also evident, although they were not uniform across subtypes: the LMM gave more negative estimates for AD (−1.32/year vs. −1.22/year), LBD (−1.18/year vs. −0.75/year), and VaD (−0.66/year vs. −0.24/year), whereas the two models were more similar for MCI (−0.20/year vs. −0.15/year) and cognitively unimpaired participants (−0.03/year vs −0.01/year), and the regression‐spline GAMM was slightly more negative for FTD (−1.19/year vs. −1.11/year). Fixed‐effect coefficient summaries from the adjusted regression‐spline GAMMs are shown in Figure S3, and full adjusted model‐fit outputs are provided in Table S3.

### 3.4 Sensitivity analyzes

Restricting NACC analyzes to observed MMSE values yielded similar qualitative subtype patterns and annualized change estimates (Table S4). Larger numerical shifts were mainly confined to smaller dementia subtypes, consistent with reduced longitudinal information after excluding MoCA‐equated visits.

In the 0‐ to 3‐year sensitivity analysis, AD remained more rapidly declining than VaD in both cohorts, although the GAMM estimates were more negative and the detailed subtype ordering differed from the 0‐ to 5‐year summary; LMM estimates were unchanged because time was modeled linearly (Table S5).

## 4 DISCUSSION

This study compared subtype‐specific MMSE trajectories across SveDem and NACC to inform cross‐cohort validation and interpretation of cognitive decline estimates in dementia. Findings from a single observational database do not always replicate elsewhere because differences in data capture, population characteristics, and health‐care settings can contribute to between‐database heterogeneity.^20^ SveDem and NACC provide a useful contrast: SveDem reflects routine clinical dementia care in Sweden, whereas NACC reflects standardized research follow‐up across U.S. Alzheimer's Disease Research Centers.^6^, ^8^, ^21^ Despite these structural differences, the relative ordering of estimated decline was broadly similar across the shared subtypes, particularly the faster decline in AD than VaD, while absolute estimates and the patterns for FTD and LBD differed. Together, these shared and cohort‐specific findings support their value as complementary resources for dementia epidemiology.

Subtype‐specific annualized MMSE decline estimates were approximately 1 point/year in the dementia groups. These estimates were lower than those reported in some previous studies, including 3.3 points/year in a meta‐analysis of Alzheimer's disease^5^ and 1.9–2.3 points/year in longitudinal Alzheimer's disease cohorts.^22^, ^23^ However, those studies primarily examined Alzheimer's disease‐focused samples, whereas the present analysis included broader real‐world dementia cohorts with more heterogeneous case‐mix. AD showed faster annualized MMSE decline than VaD in both cohorts, consistent with previous longitudinal studies.^24^, ^25^ This difference should not be interpreted as universal. Prior work suggests that relative decline between AD and vascular or cerebrovascular dementia may vary with age, with AD declining faster before 80 years of age but vascular‐related dementia progressing more rapidly in older patients.^25^ VaD may also show more domain‐specific deterioration, particularly in attention and executive function, rather than uniformly steeper decline on global cognitive measures.^2^ This may partly explain why AD showed consistently faster global MMSE decline than VaD in this MMSE‐based analysis.

For FTD and LBD, annualized MMSE decline was less consistent across cohorts. FTD showed relatively steep decline, particularly in NACC, whereas LBD estimates differed more between databases. Prior longitudinal studies have reported variable rates of cognitive decline in DLB and PDD.^26^, ^27^, ^28^ For FTD, prior findings are also inconsistent, partly because decline varies across clinical phenotypes and because MMSE may be less sensitive to the executive, behavioral, and language‐predominant deficits that characterize FTD.^1^, ^29^, ^30^ Smaller subtype‐specific sample sizes, particularly for FTD and LBD, may also have contributed to greater cross‐cohort variability.

Although MMSE is widely used in dementia research and clinical practice, repeated assessments in real‐world cohorts are often sparse and irregular. MMSE is also a bounded psychometric measure with ceiling and floor effects and variable sensitivity to change, making simple linear assumptions problematic.^10^, ^31^ Methodological work has shown that standard linear mixed models applied to crude cognitive test scores can produce biased estimates when the underlying trajectory is curvilinear.^10^, ^31^ This issue is relevant here because follow‐up remained limited despite requiring at least two assessments, with a mean of 2.9 visits in SveDem and 4.5 visits in NACC and median observation windows of 1.9 and 3.2 years, respectively. Under such sparse follow‐up, annualized decline may become sensitive to model specification, consistent with the greater divergence between GAMM‐ and LMM‐based estimates in SveDem. Prior nonlinear MMSE trajectory studies similarly support the view that apparent decline rates depend on both cohort composition and the assumed time function.^32^

### 4.1 Strengths and limitations

This study has several strengths. It used two of the world's largest longitudinal dementia data resources, representing complementary routine‐care and research settings, and applied a harmonized analytic pipeline across cohorts. The large sample size enabled subtype‐specific trajectory comparison, while the use of both regression‐spline GAMM and LMM allowed assessment of model dependence in estimated decline.

Several limitations should also be noted. First, NACC analyzes combined observed MMSE with MoCA‐equated MMSE values to improve longitudinal coverage; although MMSE‐only sensitivity analyzes supported the main qualitative findings, score conversion may have introduced measurement uncertainty.^16^ Second, dementia subtypes were clinically defined rather than biomarker‐confirmed, and diagnostic procedures differed between cohorts. Third, follow‐up was sparse and irregular, particularly in SveDem, which may have affected long‐horizon estimates and increased sensitivity to model specification.^10^, ^31^, ^32^ Correspondingly, the 0‐ to 3‐ and 0‐ to 5‐year GAMM summaries differed in magnitude and detailed subtype ordering, although AD declined faster than VaD in both cohorts over both intervals. Fourth, informative attrition due to faster decline, frailty, institutionalization, or death may have attenuated estimated decline at later follow‐up.^9^ Although assessments less than 90 days apart were excluded, repeated MMSE administration may still have introduced modest retest effects.^33^ Finally, cohort‐specific recruitment and selection processes limit direct interpretation of absolute MMSE slopes as universal disease‐course estimates.^20^

Taken together, these findings provide reference patterns for future studies using SveDem, NACC or other longitudinal dementia cohorts. For analyzes centered on AD and relative subtype patterns, the consistency across databases supports their use as external comparators. However, absolute long‐horizon estimates were more sensitive to follow‐up structure, attrition, and model specification. The main contribution of this study is therefore not to establish a universal estimate of MMSE decline, but to show that cross‐cohort comparisons can identify which trajectory features appear reproducible and which remain contingent on data structure and modeling assumptions.

## 5 CONCLUSIONS

Relative subtype‐specific MMSE decline patterns were broadly reproducible across SveDem and NACC, whereas absolute long‐horizon estimates were more sensitive to follow‐up structure and model specification. Cross‐cohort comparisons can help distinguish robust features of cognitive decline from estimates that depend on data structure. These reference patterns may inform future dementia epidemiology studies using MMSE trajectories to compare subtypes, examine clinical factors or evaluate treatment‐related associations.

## AUTHOR CONTRIBUTIONS

Xinqi Bao and Sara Garcia Ptacek conceived and designed the study. Xinqi Bao performed data processing, statistical analyzes, visualization, and drafted the manuscript. Sara Garcia Ptacek supervised the study and contributed to study design, interpretation of findings, and manuscript revision. Maria Eriksdotter contributed to study conception, clinical interpretation, and critical revision of the manuscript. Saikat Chatterjee contributed to methodological interpretation and critical revision of the manuscript. Belén García Pascual, Eirik Blakstad, Jakob Norgren, Tamar Abzhandadze, Nanbo Zhu, Minh Tuan Hoang, Martina Scolamiero, and Hong Xu contributed to data interpretation, methodological discussion, and critical revision of the manuscript. All authors read and approved the final manuscript.

## CONFLICT OF INTEREST STATEMENT

Maria Eriksdotter has served as a consultant for BioArctic AB, Roche, Eli Lilly, Biogen/Eisai, and Novo Nordisk, and has given lectures at symposia sponsored by Roche and BioArctic/Eisai. The other authors declare no conflicts of interest. Author disclosures are available in the Supporting Information.

## ETHICAL APPROVAL

The study was approved by the Swedish Ethical Review Authority (2024‐08463‐01). In accordance with Swedish regulations for quality registries, participants registered in SveDem are informed about registration and their right to opt out. NACC data were collected under protocols approved by the relevant institutional review boards at contributing Alzheimer's Disease Research Centers, with written informed consent obtained locally.

## Supporting information




Supporting Information

Supporting Information

## Data Availability

Owing to Swedish data protection regulations and registry access requirements, the individual‐level data used in this study cannot be shared by the authors. Data access may be requested from SveDem and NACC, subject to the necessary ethical approvals, data‐use agreements, and respective registry requirements for data access and confidentiality.
