# Supplementary material for: Mini‐Mental State Examination trajectories across dementia subtypes in the Swedish Registry for Cognitive/Dementia Disorders and U.S. National Alzheimer's Coordinating Center
Source: Alzheimers Dement (Amst). 2026 Oct 30;18(4):e70531. doi: 10.1002/dad2.70531 (PMC13722606; doi:10.1002/dad2.70531)
Supplement: Supplementary file 1 [file DAD2-18-e70531-s001.pdf]

ICMJE DISCLOSURE FORM

**Date:**	1/9/2026
**Your Name:**	Xinqi Bao
**Manuscript Title:**	Mini-Mental State Examination Trajectories Across Dementia Subtypes in the Swedish Registry for Cognitive/Dementia Disorders and U.S. National Alzheimer's Coordinating Center
**Manuscript Number (if known):**	DADM-D-26-00540

In the interest of transparency, we ask you to disclose all relationships/activities/interests listed below that are related to the content of your manuscript. "Related" means any relation with for-profit or not-for-profit third parties whose interests may be affected by the content of the manuscript. Disclosure represents a commitment to transparency and does not necessarily indicate a bias. If you are in doubt about whether to list a relationship/activity/interest, it is preferable that you do so. The author's relationships/activities/interests should be defined broadly. For example, if your manuscript pertains to the epidemiology of hypertension, you should declare all relationships with manufacturers of antihypertensive medication, even if that medication is not mentioned in the manuscript. In item #1 below, report all support for the work reported in this manuscript without time limit. For all other items, the time frame for disclosure is the past 36 months.

		Name all entities with whom you have this relationship or indicate none (add rows as needed)	Specifications/Comments (e.g., if payments were made to you or to your institution)
**Time frame: Since the initial planning of the work**			
**1**	All support for the present manuscript (e.g., funding, provision of study materials, medical writing, article processing charges, etc.). No time limit for this item.	☒ **None**	
**Time frame: past 36 months**			
**2**	Grants or contracts from any entity (if not indicated in item #1 above).	☒ **None**	
**3**	Royalties or licenses.	☒ **None**	

ICMJE DISCLOSURE FORM — Xinqi Bao (continued)

		Name all entities with whom you have this relationship or indicate none (add rows as needed)	Specifications/Comments (e.g., if payments were made to you or to your institution)
**4**	Consulting fees.	☒ **None**	
**5**	Payment or honoraria for lectures, presentations, speakers bureaus, manuscript writing or educational events.	☒ **None**	
**6**	Payment for expert testimony.	☒ **None**	
**7**	Support for attending meetings and/or travel.	☒ **None**	
**8**	Patents planned, issued or pending.	☒ **None**	

		Name all entities with whom you have this relationship or indicate none (add rows as needed)	Specifications/Comments (e.g., if payments were made to you or to your institution)
**9**	Participation on a Data Safety Monitoring Board or Advisory Board.	☒ **None**	
**10**	Leadership or fiduciary role in other board, society, committee or advocacy group, paid or unpaid.	☒ **None**	
**11**	Stock or stock options.	☒ **None**	
**12**	Receipt of equipment, materials, drugs, medical writing, gifts or other services.	☒ **None**	
**13**	Other financial or non-financial interests.	☒ **None**	

Please place an "X" next to the following statement to indicate your agreement:

☒ I certify that I have answered every question and have not altered the wording of any of the questions on this form.

ICMJE DISCLOSURE FORM

**Date:**	2/9/2026
**Your Name:**	Belén García Pascual
**Manuscript Title:**	Mini-Mental State Examination Trajectories Across Dementia Subtypes in the Swedish Registry for Cognitive/Dementia Disorders and U.S. National Alzheimer's Coordinating Center
**Manuscript Number (if known):**	Click or tap here to enter text.

In the interest of transparency, we ask you to disclose all relationships/activities/interests listed below that are related to the content of your manuscript. "Related" means any relation with for-profit or not-for-profit third parties whose interests may be affected by the content of the manuscript. Disclosure represents a commitment to transparency and does not necessarily indicate a bias. If you are in doubt about whether to list a relationship/activity/interest, it is preferable that you do so. The author's relationships/activities/interests should be defined broadly. For example, if your manuscript pertains to the epidemiology of hypertension, you should declare all relationships with manufacturers of antihypertensive medication, even if that medication is not mentioned in the manuscript. In item #1 below, report all support for the work reported in this manuscript without time limit. For all other items, the time frame for disclosure is the past 36 months.

		Name all entities with whom you have this relationship or indicate none (add rows as needed)	Specifications/Comments (e.g., if payments were made to you or to your institution)
**Time frame: Since the initial planning of the work**			
**1**	All support for the present manuscript (e.g., funding, provision of study materials, medical writing, article processing charges, etc.). No time limit for this item.	☒ **None**	
**Time frame: past 36 months**			
**2**	Grants or contracts from any entity (if not indicated in item #1 above).	☒ **None**	
**3**	Royalties or licenses.	☒ **None**	

		Name all entities with whom you have this relationship or indicate none (add rows as needed)	Specifications/Comments (e.g., if payments were made to you or to your institution)
**4**	Consulting fees.	☒ **None**	
**5**	Payment or honoraria for lectures, presentations, speakers bureaus, manuscript writing or educational events.	☒ **None**	
**6**	Payment for expert testimony.	☒ **None**	
**7**	Support for attending meetings and/or travel.	☒ **None**	
**8**	Patents planned, issued or pending.	☒ **None**	

		Name all entities with whom you have this relationship or indicate none (add rows as needed)	Specifications/Comments (e.g., if payments were made to you or to your institution)
**9**	Participation on a Data Safety Monitoring Board or Advisory Board.	☒ **None**	
**10**	Leadership or fiduciary role in other board, society, committee or advocacy group, paid or unpaid.	☒ **None**	
**11**	Stock or stock options.	☒ **None**	
**12**	Receipt of equipment, materials, drugs, medical writing, gifts or other services.	☒ **None**	
**13**	Other financial or non-financial interests.	☒ **None**	

Please place an "X" next to the following statement to indicate your agreement:

☒ I certify that I have answered every question and have not altered the wording of any of the questions on this form.

ICMJE DISCLOSURE FORM

**Date:**	9/1/2021
**Your Name:**	Eirik Gustav Blakstad
**Manuscript Title:**	Mini-Mental State Examination Trajectories Across Dementia Subtypes in the Swedish Registry for Cognitive/Dementia Disorders and U.S. National Alzheimer's Coordinating Center
**Manuscript Number (if known):**	DADM-D-26-00540

In the interest of transparency, we ask you to disclose all relationships/activities/interests listed below that are related to the content of your manuscript. "Related" means any relation with for-profit or not-for-profit third parties whose interests may be affected by the content of the manuscript. Disclosure represents a commitment to transparency and does not necessarily indicate a bias. If you are in doubt about whether to list a relationship/activity/interest, it is preferable that you do so. The author's relationships/activities/interests should be defined broadly. For example, if your manuscript pertains to the epidemiology of hypertension, you should declare all relationships with manufacturers of antihypertensive medication, even if that medication is not mentioned in the manuscript. In item #1 below, report all support for the work reported in this manuscript without time limit. For all other items, the time frame for disclosure is the past 36 months.

		Name all entities with whom you have this relationship or indicate none (add rows as needed)	Specifications/Comments (e.g., if payments were made to you or to your institution)
**Time frame: Since the initial planning of the work**			
**1**	All support for the present manuscript (e.g., funding, provision of study materials, medical writing, article processing charges, etc.). No time limit for this item.	☒ **None**	
**Time frame: past 36 months**			
**2**	Grants or contracts from any entity (if not indicated in item #1 above).	☒ **None**	
**3**	Royalties or licenses.	☒ **None**	

		Name all entities with whom you have this relationship or indicate none (add rows as needed)	Specifications/Comments (e.g., if payments were made to you or to your institution)
**4**	Consulting fees.	☒ **None**	
**5**	Payment or honoraria for lectures, presentations, speakers bureaus, manuscript writing or educational events.	☒ **None**	
**6**	Payment for expert testimony.	☒ **None**	
**7**	Support for attending meetings and/or travel.	☒ **None**	
**8**	Patents planned, issued or pending.	☒ **None**	

		Name all entities with whom you have this relationship or indicate none (add rows as needed)	Specifications/Comments (e.g., if payments were made to you or to your institution)
**9**	Participation on a Data Safety Monitoring Board or Advisory Board.	☒ **None**	
**10**	Leadership or fiduciary role in other board, society, committee or advocacy group, paid or unpaid.	☒ **None**	
**11**	Stock or stock options.	☒ **None**	
**12**	Receipt of equipment, materials, drugs, medical writing, gifts or other services.	☒ **None**	
**13**	Other financial or non-financial interests.	☒ **None**	

Please place an "X" next to the following statement to indicate your agreement:

☒ I certify that I have answered every question and have not altered the wording of any of the questions on this form.

ICMJE DISCLOSURE FORM

**Date:**	Septemeber 2, 2026
**Your Name:**	Jakob Norgren
**Manuscript Title:**	Mini-Mental State Examination Trajectories Across Dementia Subtypes in the Swedish Registry for Cognitive/Dementia Disorders and U.S. National Alzheimer's Coordinating Center
**Manuscript Number (if known):**	DADM-D-26-00540

In the interest of transparency, we ask you to disclose all relationships/activities/interests listed below that are related to the content of your manuscript. "Related" means any relation with for-profit or not-for-profit third parties whose interests may be affected by the content of the manuscript. Disclosure represents a commitment to transparency and does not necessarily indicate a bias. If you are in doubt about whether to list a relationship/activity/interest, it is preferable that you do so. The author's relationships/activities/interests should be defined broadly. For example, if your manuscript pertains to the epidemiology of hypertension, you should declare all relationships with manufacturers of antihypertensive medication, even if that medication is not mentioned in the manuscript. In item #1 below, report all support for the work reported in this manuscript without time limit. For all other items, the time frame for disclosure is the past 36 months.

		Name all entities with whom you have this relationship or indicate none (add rows as needed)	Specifications/Comments (e.g., if payments were made to you or to your institution)
**Time frame: Since the initial planning of the work**			
**1**	All support for the present manuscript (e.g., funding, provision of study materials, medical writing, article processing charges, etc.). No time limit for this item.	☒ **None**	
**Time frame: past 36 months**			
**2**	Grants or contracts from any entity (if not indicated in item #1 above).	☒ **None**	
**3**	Royalties or licenses.	☒ **None**	

ICMJE DISCLOSURE FORM — Jakob Norgren (continued)

		Name all entities with whom you have this relationship or indicate none (add rows as needed)	Specifications/Comments (e.g., if payments were made to you or to your institution)
**4**	Consulting fees.	☒ **None**	
**5**	Payment or honoraria for lectures, presentations, speakers bureaus, manuscript writing or educational events.	☒ **None**	
**6**	Payment for expert testimony.	☒ **None**	
**7**	Support for attending meetings and/or travel.	☒ **None**	
**8**	Patents planned, issued or pending.	☒ **None**	

		Name all entities with whom you have this relationship or indicate none (add rows as needed)	Specifications/Comments (e.g., if payments were made to you or to your institution)
**9**	Participation on a Data Safety Monitoring Board or Advisory Board.	☒ **None**	
**10**	Leadership or fiduciary role in other board, society, committee or advocacy group, paid or unpaid.	☒ **None**	
**11**	Stock or stock options.	☒ **None**	
**12**	Receipt of equipment, materials, drugs, medical writing, gifts or other services.	☒ **None**	
**13**	Other financial or non-financial interests.	☒ **None**	

Please place an "X" next to the following statement to indicate your agreement:

☒ I certify that I have answered every question and have not altered the wording of any of the questions on this form.

ICMJE DISCLOSURE FORM

**Date:**	9/1/2026
**Your Name:**	Tamar Abzhandadze
**Manuscript Title:**	Mini-Mental State Examination Trajectories Across Dementia Subtypes in the Swedish Registry for Cognitive/Dementia Disorders and U.S. National Alzheimer's Coordinating Center
**Manuscript Number (if known):**	DADM-D-26-00540

In the interest of transparency, we ask you to disclose all relationships/activities/interests listed below that are related to the content of your manuscript. "Related" means any relation with for-profit or not-for-profit third parties whose interests may be affected by the content of the manuscript. Disclosure represents a commitment to transparency and does not necessarily indicate a bias. If you are in doubt about whether to list a relationship/activity/interest, it is preferable that you do so. The author's relationships/activities/interests should be defined broadly. For example, if your manuscript pertains to the epidemiology of hypertension, you should declare all relationships with manufacturers of antihypertensive medication, even if that medication is not mentioned in the manuscript. In item #1 below, report all support for the work reported in this manuscript without time limit. For all other items, the time frame for disclosure is the past 36 months.

		Name all entities with whom you have this relationship or indicate none (add rows as needed)	Specifications/Comments (e.g., if payments were made to you or to your institution)
**Time frame: Since the initial planning of the work**			
**1**	All support for the present manuscript (e.g., funding, provision of study materials, medical writing, article processing charges, etc.). No time limit for this item.	☒ **None**	
**Time frame: past 36 months**			
**2**	Grants or contracts from any entity (if not indicated in item #1 above).	☒ **None**	
**3**	Royalties or licenses.	☒ **None**	

		Name all entities with whom you have this relationship or indicate none (add rows as needed)	Specifications/Comments (e.g., if payments were made to you or to your institution)
**4**	Consulting fees.	☒ **None**	
**5**	Payment or honoraria for lectures, presentations, speakers bureaus, manuscript writing or educational events.	☒ **None**	
**6**	Payment for expert testimony.	☒ **None**	
**7**	Support for attending meetings and/or travel.	☒ **None**	
**8**	Patents planned, issued or pending.	☒ **None**	

ICMJE DISCLOSURE FORM — Tamar Abzhandadze (continued)

		Name all entities with whom you have this relationship or indicate none (add rows as needed)	Specifications/Comments (e.g., if payments were made to you or to your institution)
**9**	Participation on a Data Safety Monitoring Board or Advisory Board.	☒ **None**	
**10**	Leadership or fiduciary role in other board, society, committee or advocacy group, paid or unpaid.	☒ **None**	
**11**	Stock or stock options.	☒ **None**	
**12**	Receipt of equipment, materials, drugs, medical writing, gifts or other services.	☒ **None**	
**13**	Other financial or non-financial interests.	☒ **None**	

Please place an "X" next to the following statement to indicate your agreement:

☒ I certify that I have answered every question and have not altered the wording of any of the questions on this form.

ICMJE DISCLOSURE FORM

**Date:**	9/1/2026
**Your Name:**	Nanbo Zhu
**Manuscript Title:**	Mini-Mental State Examination Trajectories Across Dementia Subtypes in the Swedish Registry for Cognitive/Dementia Disorders and U.S. National Alzheimer's Coordinating Center
**Manuscript Number (if known):**	DADM-D-26-00540

In the interest of transparency, we ask you to disclose all relationships/activities/interests listed below that are related to the content of your manuscript. "Related" means any relation with for-profit or not-for-profit third parties whose interests may be affected by the content of the manuscript. Disclosure represents a commitment to transparency and does not necessarily indicate a bias. If you are in doubt about whether to list a relationship/activity/interest, it is preferable that you do so. The author's relationships/activities/interests should be defined broadly. For example, if your manuscript pertains to the epidemiology of hypertension, you should declare all relationships with manufacturers of antihypertensive medication, even if that medication is not mentioned in the manuscript. In item #1 below, report all support for the work reported in this manuscript without time limit. For all other items, the time frame for disclosure is the past 36 months.

		Name all entities with whom you have this relationship or indicate none (add rows as needed)	Specifications/Comments (e.g., if payments were made to you or to your institution)
**Time frame: Since the initial planning of the work**			
**1**	All support for the present manuscript (e.g., funding, provision of study materials, medical writing, article processing charges, etc.). No time limit for this item.	☒ **None**	
**Time frame: past 36 months**			
**2**	Grants or contracts from any entity (if not indicated in item #1 above).	☒ **None**	
**3**	Royalties or licenses.	☒ **None**	

		Name all entities with whom you have this relationship or indicate none (add rows as needed)	Specifications/Comments (e.g., if payments were made to you or to your institution)
**4**	Consulting fees.	☒ **None**	
**5**	Payment or honoraria for lectures, presentations, speakers bureaus, manuscript writing or educational events.	☒ **None**	
**6**	Payment for expert testimony.	☒ **None**	
**7**	Support for attending meetings and/or travel.	☒ **None**	
**8**	Patents planned, issued or pending.	☒ **None**	

ICMJE DISCLOSURE FORM — Nanbo Zhu (continued)

		Name all entities with whom you have this relationship or indicate none (add rows as needed)	Specifications/Comments (e.g., if payments were made to you or to your institution)
**9**	Participation on a Data Safety Monitoring Board or Advisory Board.	☒ **None**	
**10**	Leadership or fiduciary role in other board, society, committee or advocacy group, paid or unpaid.	☒ **None**	
**11**	Stock or stock options.	☒ **None**	
**12**	Receipt of equipment, materials, drugs, medical writing, gifts or other services.	☒ **None**	
**13**	Other financial or non-financial interests.	☒ **None**	

Please place an "X" next to the following statement to indicate your agreement:

☒ I certify that I have answered every question and have not altered the wording of any of the questions on this form.

ICMJE DISCLOSURE FORM

**Date:**	9/1/2026
**Your Name:**	Minh Tuan Hoang
**Manuscript Title:**	Mini-Mental State Examination Trajectories Across Dementia Subtypes in the Swedish Registry for Cognitive/Dementia Disorders and U.S. National Alzheimer's Coordinating Center
**Manuscript Number (if known):**	DADM-D-26-00540

In the interest of transparency, we ask you to disclose all relationships/activities/interests listed below that are related to the content of your manuscript. "Related" means any relation with for-profit or not-for-profit third parties whose interests may be affected by the content of the manuscript. Disclosure represents a commitment to transparency and does not necessarily indicate a bias. If you are in doubt about whether to list a relationship/activity/interest, it is preferable that you do so. The author's relationships/activities/interests should be defined broadly. For example, if your manuscript pertains to the epidemiology of hypertension, you should declare all relationships with manufacturers of antihypertensive medication, even if that medication is not mentioned in the manuscript. In item #1 below, report all support for the work reported in this manuscript without time limit. For all other items, the time frame for disclosure is the past 36 months.

		Name all entities with whom you have this relationship or indicate none (add rows as needed)	Specifications/Comments (e.g., if payments were made to you or to your institution)
**Time frame: Since the initial planning of the work**			
**1**	All support for the present manuscript (e.g., funding, provision of study materials, medical writing, article processing charges, etc.). No time limit for this item.	☒ **None**	
**Time frame: past 36 months**			
**2**	Grants or contracts from any entity (if not indicated in item #1 above).	☒ **None**	
**3**	Royalties or licenses.	☒ **None**	

ICMJE DISCLOSURE FORM — Minh Tuan Hoang (continued)

		Name all entities with whom you have this relationship or indicate none (add rows as needed)	Specifications/Comments (e.g., if payments were made to you or to your institution)
**4**	Consulting fees.	☒ **None**	
**5**	Payment or honoraria for lectures, presentations, speakers bureaus, manuscript writing or educational events.	☒ **None**	
**6**	Payment for expert testimony.	☒ **None**	
**7**	Support for attending meetings and/or travel.	☒ **None**	
**8**	Patents planned, issued or pending.	☒ **None**	

ICMJE DISCLOSURE FORM — Minh Tuan Hoang (continued)

		Name all entities with whom you have this relationship or indicate none (add rows as needed)	Specifications/Comments (e.g., if payments were made to you or to your institution)
**9**	Participation on a Data Safety Monitoring Board or Advisory Board.	☒ **None**	
**10**	Leadership or fiduciary role in other board, society, committee or advocacy group, paid or unpaid.	☒ **None**	
**11**	Stock or stock options.	☒ **None**	
**12**	Receipt of equipment, materials, drugs, medical writing, gifts or other services.	☒ **None**	
**13**	Other financial or non-financial interests.	☒ **None**	

Please place an "X" next to the following statement to indicate your agreement:

☒ I certify that I have answered every question and have not altered the wording of any of the questions on this form.

ICMJE DISCLOSURE FORM

**Date:**	4 September 2026
**Your Name:**	Saikat Chatterjee
**Manuscript Title:**	Mini-Mental State Examination Trajectories Across Dementia Subtypes in the Swedish Registry for Cognitive/Dementia Disorders and U.S. National Alzheimer's Coordinating Center
**Manuscript Number (if known):**	DADM-D-26-00540

In the interest of transparency, we ask you to disclose all relationships/activities/interests listed below that are related to the content of your manuscript. "Related" means any relation with for-profit or not-for-profit third parties whose interests may be affected by the content of the manuscript. Disclosure represents a commitment to transparency and does not necessarily indicate a bias. If you are in doubt about whether to list a relationship/activity/interest, it is preferable that you do so. The author's relationships/activities/interests should be defined broadly. For example, if your manuscript pertains to the epidemiology of hypertension, you should declare all relationships with manufacturers of antihypertensive medication, even if that medication is not mentioned in the manuscript. In item #1 below, report all support for the work reported in this manuscript without time limit. For all other items, the time frame for disclosure is the past 36 months.

		Name all entities with whom you have this relationship or indicate none (add rows as needed)	Specifications/Comments (e.g., if payments were made to you or to your institution)
**Time frame: Since the initial planning of the work**			
**1**	All support for the present manuscript (e.g., funding, provision of study materials, medical writing, article processing charges, etc.). No time limit for this item.	☒ **None**	
**Time frame: past 36 months**			
**2**	Grants or contracts from any entity (if not indicated in item #1 above).	☒ **None**	
**3**	Royalties or licenses.	☒ **None**	

		Name all entities with whom you have this relationship or indicate none (add rows as needed)	Specifications/Comments (e.g., if payments were made to you or to your institution)
**4**	Consulting fees.	☒ **None**	
**5**	Payment or honoraria for lectures, presentations, speakers bureaus, manuscript writing or educational events.	☒ **None**	
**6**	Payment for expert testimony.	☒ **None**	
**7**	Support for attending meetings and/or travel.	☒ **None**	
**8**	Patents planned, issued or pending.	☒ **None**	

		Name all entities with whom you have this relationship or indicate none (add rows as needed)	Specifications/Comments (e.g., if payments were made to you or to your institution)
**9**	Participation on a Data Safety Monitoring Board or Advisory Board.	☒ **None**	
**10**	Leadership or fiduciary role in other board, society, committee or advocacy group, paid or unpaid.	☒ **None**	
**11**	Stock or stock options.	☒ **None**	
**12**	Receipt of equipment, materials, drugs, medical writing, gifts or other services.	☒ **None**	
**13**	Other financial or non-financial interests.	☒ **None**	

Please place an "X" next to the following statement to indicate your agreement:

☒ I certify that I have answered every question and have not altered the wording of any of the questions on this form.

ICMJE DISCLOSURE FORM

**Date:**	4 September 2026
**Your Name:**	Martina Scolamiero
**Manuscript Title:**	Mini-Mental State Examination Trajectories Across Dementia Subtypes in the Swedish Registry for Cognitive/Dementia Disorders and U.S. National Alzheimer's Coordinating Center
**Manuscript Number (if known):**	DADM-D-26-00540

In the interest of transparency, we ask you to disclose all relationships/activities/interests listed below that are related to the content of your manuscript. "Related" means any relation with for-profit or not-for-profit third parties whose interests may be affected by the content of the manuscript. Disclosure represents a commitment to transparency and does not necessarily indicate a bias. If you are in doubt about whether to list a relationship/activity/interest, it is preferable that you do so. The author's relationships/activities/interests should be defined broadly. For example, if your manuscript pertains to the epidemiology of hypertension, you should declare all relationships with manufacturers of antihypertensive medication, even if that medication is not mentioned in the manuscript. In item #1 below, report all support for the work reported in this manuscript without time limit. For all other items, the time frame for disclosure is the past 36 months.

		Name all entities with whom you have this relationship or indicate none (add rows as needed)	Specifications/Comments (e.g., if payments were made to you or to your institution)
**Time frame: Since the initial planning of the work**			
**1**	All support for the present manuscript (e.g., funding, provision of study materials, medical writing, article processing charges, etc.). No time limit for this item.	☒ **None**	
**Time frame: past 36 months**			
**2**	Grants or contracts from any entity (if not indicated in item #1 above).	☒ **None**	
**3**	Royalties or licenses.	☒ **None**	

		Name all entities with whom you have this relationship or indicate none (add rows as needed)	Specifications/Comments (e.g., if payments were made to you or to your institution)
**4**	Consulting fees.	☒ **None**	
**5**	Payment or honoraria for lectures, presentations, speakers bureaus, manuscript writing or educational events.	☒ **None**	
**6**	Payment for expert testimony.	☒ **None**	
**7**	Support for attending meetings and/or travel.	☒ **None**	
**8**	Patents planned, issued or pending.	☒ **None**	

ICMJE DISCLOSURE FORM — Martina Scolamiero (continued)

		Name all entities with whom you have this relationship or indicate none (add rows as needed)	Specifications/Comments (e.g., if payments were made to you or to your institution)
**9**	Participation on a Data Safety Monitoring Board or Advisory Board.	☒ **None**	
**10**	Leadership or fiduciary role in other board, society, committee or advocacy group, paid or unpaid.	☒ **None**	
**11**	Stock or stock options.	☒ **None**	
**12**	Receipt of equipment, materials, drugs, medical writing, gifts or other services.	☒ **None**	
**13**	Other financial or non-financial interests.	☒ **None**	

Please place an "X" next to the following statement to indicate your agreement:

☒ I certify that I have answered every question and have not altered the wording of any of the questions on this form.

ICMJE DISCLOSURE FORM

**Date:**	9/4/2026
**Your Name:**	Maria Eriksdotter
**Manuscript Title:**	Mini-Mental State Examination Trajectories Across Dementia Subtypes in the Swedish Registry for Cognitive/Dementia Disorders and U.S. National Alzheimer's Coordinating Center
**Manuscript Number (if known):**	DADM-D-26-00540

In the interest of transparency, we ask you to disclose all relationships/activities/interests listed below that are related to the content of your manuscript. "Related" means any relation with for-profit or not-for-profit third parties whose interests may be affected by the content of the manuscript. Disclosure represents a commitment to transparency and does not necessarily indicate a bias. If you are in doubt about whether to list a relationship/activity/interest, it is preferable that you do so. The author's relationships/activities/interests should be defined broadly. For example, if your manuscript pertains to the epidemiology of hypertension, you should declare all relationships with manufacturers of antihypertensive medication, even if that medication is not mentioned in the manuscript. In item #1 below, report all support for the work reported in this manuscript without time limit. For all other items, the time frame for disclosure is the past 36 months.

		Name all entities with whom you have this relationship or indicate none (add rows as needed)	Specifications/Comments (e.g., if payments were made to you or to your institution)
**Time frame: Since the initial planning of the work**			
**1**	All support for the present manuscript (e.g., funding, provision of study materials, medical writing, article processing charges, etc.). No time limit for this item.	☒ **None**	
**Time frame: past 36 months**			
**2**	Grants or contracts from any entity (if not indicated in item #1 above).	☐ **None**	
		Alzecure pharma for experimental project in 2024, finished and reported)	
**3**	Royalties or licenses.	☒ **None**	

		Name all entities with whom you have this relationship or indicate none (add rows as needed)	Specifications/Comments (e.g., if payments were made to you or to your institution)
**4**	Consulting fees.	☐ **None**	
		Consulting at Swedish National Alzheimer Disease advisory board meeting Bioarctic/Eisai 20230919.	
		Expert meeting with NWE Alzheimer's Disease Advisory Board, Novonordisk A/S, 20241126.	
		Expert meeting with Eli Lilly Stockholm 20250530	
		Expert meeting Bioarctic/Eisai, Stockholm 20260202	
**5**	Payment or honoraria for lectures, presentations, speakers bureaus, manuscript writing or educational events.	☐ **None**	
		Lecture at symposium Nordic ALzheimer meeting, Organized by Eisai/Bioarctic 5-6 sept 2025	
		Lecture on dementia at Elderly care conference for staff and leadership in municipalities, organized by Gothia Konferens, Oct 16 2025.	
**6**	Payment for expert testimony.	☒ **None**	
**7**	Support for attending meetings and/or travel.	☒ **None**	
**8**	Patents planned, issued or pending.	☒ **None**	

ICMJE DISCLOSURE FORM — Maria Eriksdotter (continued)

		Name all entities with whom you have this relationship or indicate none (add rows as needed)	Specifications/Comments (e.g., if payments were made to you or to your institution)
**9**	Participation on a Data Safety Monitoring Board or Advisory Board.	☐ **None**	
		See above)	
**10**	Leadership or fiduciary role in other board, society, committee or advocacy group, paid or unpaid.	☐ **None**	
		Member of the Boards of Silviahemmet foundation, the Swedish Dementia Centre, the foundation Borgerskapets Enkehus och Gubbhus,	
**11**	Stock or stock options.	☐ **None**	
		Gilead, Lundbeck	
**12**	Receipt of equipment, materials, drugs, medical writing, gifts or other services.	☒ **None**	
**13**	Other financial or non-financial interests.	☒ **None**	

Please place an "X" next to the following statement to indicate your agreement:

☒ I certify that I have answered every question and have not altered the wording of any of the questions on this form.

ICMJE DISCLOSURE FORM

**Date:**	9/1/2026
**Your Name:**	Hong Xu
**Manuscript Title:**	Mini-Mental State Examination Trajectories Across Dementia Subtypes in the Swedish Registry for Cognitive/Dementia Disorders and U.S. National Alzheimer's Coordinating Center
**Manuscript Number (if known):**	DADM-D-26-00540

In the interest of transparency, we ask you to disclose all relationships/activities/interests listed below that are related to the content of your manuscript. "Related" means any relation with for-profit or not-for-profit third parties whose interests may be affected by the content of the manuscript. Disclosure represents a commitment to transparency and does not necessarily indicate a bias. If you are in doubt about whether to list a relationship/activity/interest, it is preferable that you do so. The author's relationships/activities/interests should be defined broadly. For example, if your manuscript pertains to the epidemiology of hypertension, you should declare all relationships with manufacturers of antihypertensive medication, even if that medication is not mentioned in the manuscript. In item #1 below, report all support for the work reported in this manuscript without time limit. For all other items, the time frame for disclosure is the past 36 months.

		Name all entities with whom you have this relationship or indicate none (add rows as needed)	Specifications/Comments (e.g., if payments were made to you or to your institution)
**Time frame: Since the initial planning of the work**			
**1**	All support for the present manuscript (e.g., funding, provision of study materials, medical writing, article processing charges, etc.). No time limit for this item.	☒ **None**	
**Time frame: past 36 months**			
**2**	Grants or contracts from any entity (if not indicated in item #1 above).	☒ **None**	
**3**	Royalties or licenses.	☒ **None**	

ICMJE DISCLOSURE FORM — Hong Xu (continued)

		Name all entities with whom you have this relationship or indicate none (add rows as needed)	Specifications/Comments (e.g., if payments were made to you or to your institution)
**4**	Consulting fees.	☒ **None**	
**5**	Payment or honoraria for lectures, presentations, speakers bureaus, manuscript writing or educational events.	☒ **None**	
**6**	Payment for expert testimony.	☒ **None**	
**7**	Support for attending meetings and/or travel.	☒ **None**	
**8**	Patents planned, issued or pending.	☒ **None**	

ICMJE DISCLOSURE FORM — Hong Xu (continued)

		Name all entities with whom you have this relationship or indicate none (add rows as needed)	Specifications/Comments (e.g., if payments were made to you or to your institution)
**9**	Participation on a Data Safety Monitoring Board or Advisory Board.	☒ **None**	
**10**	Leadership or fiduciary role in other board, society, committee or advocacy group, paid or unpaid.	☒ **None**	
**11**	Stock or stock options.	☒ **None**	
**12**	Receipt of equipment, materials, drugs, medical writing, gifts or other services.	☒ **None**	
**13**	Other financial or non-financial interests.	☒ **None**	

Please place an "X" next to the following statement to indicate your agreement:

☒ I certify that I have answered every question and have not altered the wording of any of the questions on this form.

ICMJE DISCLOSURE FORM

**Date:**	8/26/2021
**Your Name:**	Sara Garcia-Ptacek
**Manuscript Title:**	Mini-Mental State Examination Trajectories Across Dementia Subtypes in the Swedish Registry for Cognitive/Dementia Disorders and U.S. National Alzheimer's Coordinating Center
**Manuscript Number (if known):**	Click or tap here to enter text.

In the interest of transparency, we ask you to disclose all relationships/activities/interests listed below that are related to the content of your manuscript. "Related" means any relation with for-profit or not-for-profit third parties whose interests may be affected by the content of the manuscript. Disclosure represents a commitment to transparency and does not necessarily indicate a bias. If you are in doubt about whether to list a relationship/activity/interest, it is preferable that you do so. The author's relationships/activities/interests should be defined broadly. For example, if your manuscript pertains to the epidemiology of hypertension, you should declare all relationships with manufacturers of antihypertensive medication, even if that medication is not mentioned in the manuscript. In item #1 below, report all support for the work reported in this manuscript without time limit. For all other items, the time frame for disclosure is the past 36 months.

		Name all entities with whom you have this relationship or indicate none (add rows as needed)	Specifications/Comments (e.g., if payments were made to you or to your institution)
**Time frame: Since the initial planning of the work**			
**1**	All support for the present manuscript (e.g., funding, provision of study materials, medical writing, article processing charges, etc.). No time limit for this item.	☐ **None**	
		Swedish Research Council, New Innovative Roads Call, Cornell Foundation, Alzheimerfoundation, Dementia Foundation, KI Central Positions, ALF, CIMED	
**Time frame: past 36 months**			
**2**	Grants or contracts from any entity (if not indicated in item #1 above).	☒ **None**	
**3**	Royalties or licenses.	☒ **None**	

		Name all entities with whom you have this relationship or indicate none (add rows as needed)	Specifications/Comments (e.g., if payments were made to you or to your institution)
**4**	Consulting fees.	☒ **None**	
**5**	Payment or honoraria for lectures, presentations, speakers bureaus, manuscript writing or educational events.	☒ **None**	
**6**	Payment for expert testimony.	☒ **None**	
**7**	Support for attending meetings and/or travel.	☒ **None**	
**8**	Patents planned, issued or pending.	☒ **None**	

ICMJE DISCLOSURE FORM — Sara Garcia-Ptacek (continued)

		Name all entities with whom you have this relationship or indicate none (add rows as needed)	Specifications/Comments (e.g., if payments were made to you or to your institution)
**9**	Participation on a Data Safety Monitoring Board or Advisory Board.	☐ **None**	
		Member of the Swedish Ethical Review Authority	
**10**	Leadership or fiduciary role in other board, society, committee or advocacy group, paid or unpaid.	☐ **None**	
		Board member Old Servants Association	
**11**	Stock or stock options.	☒ **None**	
**12**	Receipt of equipment, materials, drugs, medical writing, gifts or other services.	☒ **None**	
**13**	Other financial or non-financial interests.	☒ **None**	

Please place an "X" next to the following statement to indicate your agreement:

☒ I certify that I have answered every question and have not altered the wording of any of the questions on this form.
