# Supplementary material for: Mini‐Mental State Examination trajectories across dementia subtypes in the Swedish Registry for Cognitive/Dementia Disorders and U.S. National Alzheimer's Coordinating Center
Source: Alzheimers Dement (Amst). 2026 Oct 30;18(4):e70531. doi: 10.1002/dad2.70531 (PMC13722606; doi:10.1002/dad2.70531)
Supplement: Supplementary file 2 [file DAD2-18-e70531-s002.pdf]

Supplementary Material

Mini-Mental State Examination Trajectories Across Dementia Subtypes in the Swedish Registry for Cognitive/Dementia Disorders and U.S. National Alzheimer's Coordinating Center

Additional file 1: Supplementary methods, tables, and figures

Supplementary Methods

S1. Detailed harmonization rules for baseline covariates

Education (SveDem SUN2000 to years). Education in SveDem was recorded using SUN2000 categories and converted to years of schooling using a prespecified mapping consistent with the LISA framework. When a SUN category corresponded to an interval of years, the midpoint of that interval was assigned to obtain an approximate continuous measure of schooling. For example, SUN = 1 indicates incomplete compulsory education (defined as <9 years), for which 4.5 years was assigned as a midpoint approximation. When a category corresponded to a fixed duration (e.g., completed compulsory education at 9 years), that value was assigned directly.

Civil (marital) status mapping. Civil status in SveDem follows the LISA coding scheme. To harmonize civil status across SveDem and NACC, a two-step mapping was applied.

Table S1. Civil status harmonization: mapping of NACC MARISTAT categories to SveDem/LISA codes and analytic categories.

NACC MARISTAT	SveDem/LISA	Analytic category
1 = Married	G	Married
6 = Living as married	G	Married
2 = Widowed	Ä	Widowed
3 = Never married	OG	Single
4 = Divorced	S	Divorced
5 = Separated	S	Divorced
9 = Other/unknown/missing	–	Excluded

Comorbidity count: component conditions and data sources. The comorbidity count was defined as an unweighted sum of 11 chronic disease indicators available in both SveDem and NACC: ischemic heart disease, heart failure, stroke or transient ischemic attack, diabetes, hypertension, hypercholesterolemia, rheumatologic disease, non-metastatic cancer, metastatic cancer, HIV infection, and seizures/epilepsy. This count was loosely modeled on the Charlson comorbidity score, but was not intended to reproduce the full Charlson index because the available comorbidity information and ascertainment mechanisms differed between cohorts. In SveDem, these conditions were derived from ICD-10 diagnostic codes recorded in the Swedish National Patient Register. In NACC, they were identified using participant or informant report together with clinician documentation. The variable should therefore be interpreted as a pragmatic count of recorded comorbidity rather than as a formally harmonized or validated comorbidity index.

S2. Supplementary trajectory figures

To keep the main text focused on adjusted comparisons, unadjusted subtype-specific MMSE trajectories are provided in Figure S2. These plots allow visual comparison between crude and adjusted trajectory patterns within each database and model class. In the present data, adjustment primarily shifted baseline levels and did not materially alter the broad relative ordering of subtype-specific decline.

Figure S3 shows fixed-effect coefficients from the adjusted regression-spline GAMMs by cohort and subtype. These coefficients are presented to describe the adjusted models and covariate structure and should not be interpreted causally.

S3. Model estimation and additional summaries

Software and estimation. All models were estimated using MixedLM in statsmodels. Restricted maximum likelihood (REML) was used for parameter estimation by default. For information-criterion reporting, models were refitted using maximum likelihood (ML), because information criteria are conventionally defined for ML fits and are not directly comparable across models fitted with REML.

Adjusted predictions: reference covariate profiles. Adjusted predicted trajectories and derived summaries were evaluated at model-specific reference profiles. Continuous covariates were set to subtype-specific means in each analytic sample, and categorical covariates were set to female sex and married/cohabiting. Baseline MMSE was mean-centered within each model and set to 0 for adjusted predictions. Exact values are reported in Table S2.

Variance components and explained-variance summaries. Model outputs include the random-intercept variance (τ^2) and residual variance (σ^2). Marginal and conditional R^2 were computed following Nakagawa and Schielzeth and are reported descriptively to characterize model fit and variance partitioning.

Information criteria and log-likelihood. Log-likelihood, Akaike Information Criterion, and Bayesian Information Criterion are reported descriptively for model classes fitted to the same cohort and subtype. Lower information-criterion values indicate better relative fit within the same dataset and outcome definition.

Derived change metric and uncertainty. Average annual change over 0–5 years was computed as $[\hat{\mu}(5) - \hat{\mu}(0)]/5$, where $\hat{\mu}(t)$ denotes the fixed-effect predicted mean MMSE at year t under the model-specific reference profile. The corresponding 0–3-year value was computed as $[\hat{\mu}(3) - \hat{\mu}(0)]/3$. Confidence intervals were obtained using the delta method based on the fixed-effect covariance matrix. Negative values indicate decline.

Supplementary Results

Table S2. Model-specific reference covariate profiles used for adjusted predictions.

Database	Subtype	Baseline MMSE	Age, years	Education, years	Comorbidity count	Sex	Marital status
SveDem	AD	22.91	75.83	11.66	0.88	Female	Married/cohabiting
	Mixed dementia	22.44	79.40	11.19	1.50	Female	Married/cohabiting
	VaD	22.52	79.22	10.57	1.78	Female	Married/cohabiting
	LBD	23.14	74.63	12.25	1.16	Female	Married/cohabiting
	FTD	24.50	70.01	12.37	0.74	Female	Married/cohabiting
NACC	Cognitively unimpaired	29.05	70.15	16.03	2.33	Female	Married/cohabiting
	AD	22.62	74.57	14.75	2.07	Female	Married/cohabiting
	LBD	23.95	72.17	15.69	2.32	Female	Married/cohabiting
	FTD	23.84	64.22	15.51	1.76	Female	Married/cohabiting
	MCI	27.49	73.25	15.53	2.47	Female	Married/cohabiting
	VaD	24.50	75.82	14.71	3.39	Female	Married/cohabiting

Note. Continuous covariates were set to subtype-specific means in each fitted model. Baseline MMSE was centered before fitting; a centered value of 0 corresponds to the uncentered mean shown here. Categorical covariates were set to the listed reference categories. CI, shared comorbidity count.

Table S3. Full adjusted model outputs by cohort, subtype, and model class, including information criteria.

Database	Model	Subtype	logLik	AIC	BIC	τ^2	σ^2	R^2m	R^2c	Ann. change
SveDem	Regression-spline GAMM	AD	−92,515.39	185,066.79	185,219.52	2.998	8.063	0.570	0.687	−0.789
		FTD	−2,758.35	5,550.70	5,634.47	3.066	10.618	0.543	0.646	−0.661
		LBD	−4,615.57	9,265.14	9,358.09	2.690	9.236	0.486	0.602	−0.458
		Mixed dementia	−35,571.83	71,177.67	71,306.14	2.154	7.210	0.585	0.680	−0.654
		VaD	−27,233.25	54,500.50	54,624.18	2.402	7.736	0.554	0.660	−0.493
	LMM	AD	−95,686.47	191,396.95	191,498.77	5.999	8.456	0.566	0.746	−1.136
		FTD	−2,940.82	5,903.64	5,957.85	9.281	12.620	0.482	0.702	−0.806
		LBD	−4,735.58	9,493.17	9,553.32	4.730	9.628	0.486	0.656	−0.982
		Mixed dementia	−36,767.25	73,556.50	73,639.63	4.357	7.504	0.574	0.731	−1.065
		VaD	−28,374.78	56,771.55	56,851.58	6.018	8.020	0.533	0.733	−0.707
NACC	Regression-spline GAMM	AD	−74,186.45	148,406.90	148,546.11	6.484	11.334	0.621	0.759	−1.224
		FTD	−8,880.94	17,795.88	17,897.83	8.234	17.298	0.606	0.733	−1.194
		Cognitively unimpaired	−122,268.37	244,570.75	244,728.27	0.388	1.108	0.375	0.537	−0.011
		LBD	−5,681.30	11,396.60	11,491.30	7.682	15.071	0.517	0.680	−0.754
		MCI	−48,648.95	97,331.91	97,468.84	1.207	2.986	0.446	0.606	−0.155
		VaD	−1,382.34	–	–	6.407	10.669	0.536	0.710	−0.244
	LMM	AD	−76,329.90	152,681.80	152,771.88	11.489	11.927	0.597	0.795	−1.320
		FTD	−9,328.55	18,679.10	18,745.07	14.809	21.729	0.536	0.724	−1.109
		Cognitively unimpaired	−144,360.14	–	–	10.043	1.124	0.470	0.947	−0.034
		LBD	−5,854.33	11,730.67	11,791.95	13.772	16.080	0.498	0.729	−1.183
		MCI	−54,250.58	–	–	9.050	3.026	0.478	0.869	−0.207
		VaD	−1,455.92	–	–	17.438	10.799	0.458	0.793	−0.660

Note. τ^2 denotes random-intercept variance and σ^2 residual variance. R^2m and R^2c denote marginal and conditional explained variance. Annualized change is the model-based mean annual MMSE change over 0–5 years, calculated as $[\hat{\mu}(5) - \hat{\mu}(0)]/5$; negative values indicate decline. AIC and BIC are shown as "–" when unavailable. AD, Alzheimer's disease; FTD, frontotemporal dementia; GAMM, generalized additive mixed model; LBD, Lewy body dementias; LMM, linear mixed-effects model; MCI, mild cognitive impairment; VaD, vascular dementia.

Table S4. NACC sensitivity analysis comparing adjusted regression-spline GAMMs fitted using MMSE+MoCA-equated outcomes (primary) versus MMSE-only outcomes (sensitivity).

Subtype	Primary nsubj/nobs	τ^2	σ^2	R^2marg	R^2cond	Ann. change	MMSE-only nsubj/nobs	τ^2	σ^2	R^2marg	R^2cond	Ann. change
AD	8,306 / 26,597	6.484	11.334	0.621	0.759	−1.22	5,940 / 18,976	6.056	10.400	0.652	0.780	−1.17
FTD	1,007 / 2,973	8.234	17.298	0.606	0.733	−1.19	661 / 1,918	9.290	18.210	0.618	0.747	−1.41
Cognitively unimpaired	15,950 / 78,146	0.388	1.108	0.375	0.537	−0.011	9,173 / 41,598	0.310	0.989	0.374	0.523	−0.013
LBD	667 / 1,941	7.682	15.071	0.517	0.680	−0.754	452 / 1,270	7.347	13.390	0.580	0.729	−1.046
MCI	7,354 / 23,269	1.207	2.986	0.446	0.606	−0.155	4,220 / 12,994	0.997	2.663	0.424	0.581	−0.135
VaD	183 / 500	6.407	10.669	0.536	0.710	−0.244	127 / 338	3.469	8.653	0.651	0.751	−0.211

Note. nsubj/nobs denotes the number of unique participants and retained cognitive assessments in each subtype-specific model. The primary outcome combines observed MMSE and MoCA-equated MMSE; the sensitivity analysis retains observed MMSE only. Annualized change is the model-based mean annual MMSE change over 0–5 years; negative values indicate decline. Because NACC classification was visit-specific, participants could contribute visits to different state-specific models across follow-up.

Table S5. Sensitivity analysis comparing adjusted average annual MMSE change over 0–3 years with the primary 0–5-year estimates.

Database	Subtype	GAMM, 0–5 y (primary)	GAMM, 0–3 y (sensitivity)	LMM, 0–3 and 0–5 y
SveDem	AD	−0.79 (−0.81, −0.77)	−0.82 (−0.85, −0.79)	−1.14 (−1.16, −1.11)
	Mixed dementia	−0.65 (−0.69, −0.62)	−0.68 (−0.73, −0.64)	−1.06 (−1.10, −1.03)
	VaD	−0.49 (−0.53, −0.46)	−0.57 (−0.62, −0.52)	−0.71 (−0.75, −0.67)
	LBD	−0.46 (−0.57, −0.35)	−0.60 (−0.76, −0.45)	−0.98 (−1.11, −0.86)
	FTD	−0.66 (−0.79, −0.54)	−0.92 (−1.12, −0.73)	−0.81 (−0.96, −0.66)
NACC	AD	−1.22 (−1.25, −1.20)	−1.46 (−1.50, −1.42)	−1.32 (−1.35, −1.29)
	VaD	−0.24 (−0.46, −0.03)	−0.52 (−0.86, −0.18)	−0.66 (−0.83, −0.49)
	LBD	−0.75 (−0.87, −0.64)	−0.89 (−1.07, −0.72)	−1.18 (−1.30, −1.07)
	FTD	−1.19 (−1.29, −1.10)	−1.51 (−1.65, −1.37)	−1.11 (−1.21, −1.01)

Note. Values are adjusted average annual MMSE change in points/year (95% confidence interval); negative values indicate decline. The 0–3- and 0–5-year GAMM summaries were calculated from the same fitted models and reference covariate profiles. Because the LMM assumes linear time, its annualized estimate is identical over the two windows.

Supplementary Figures

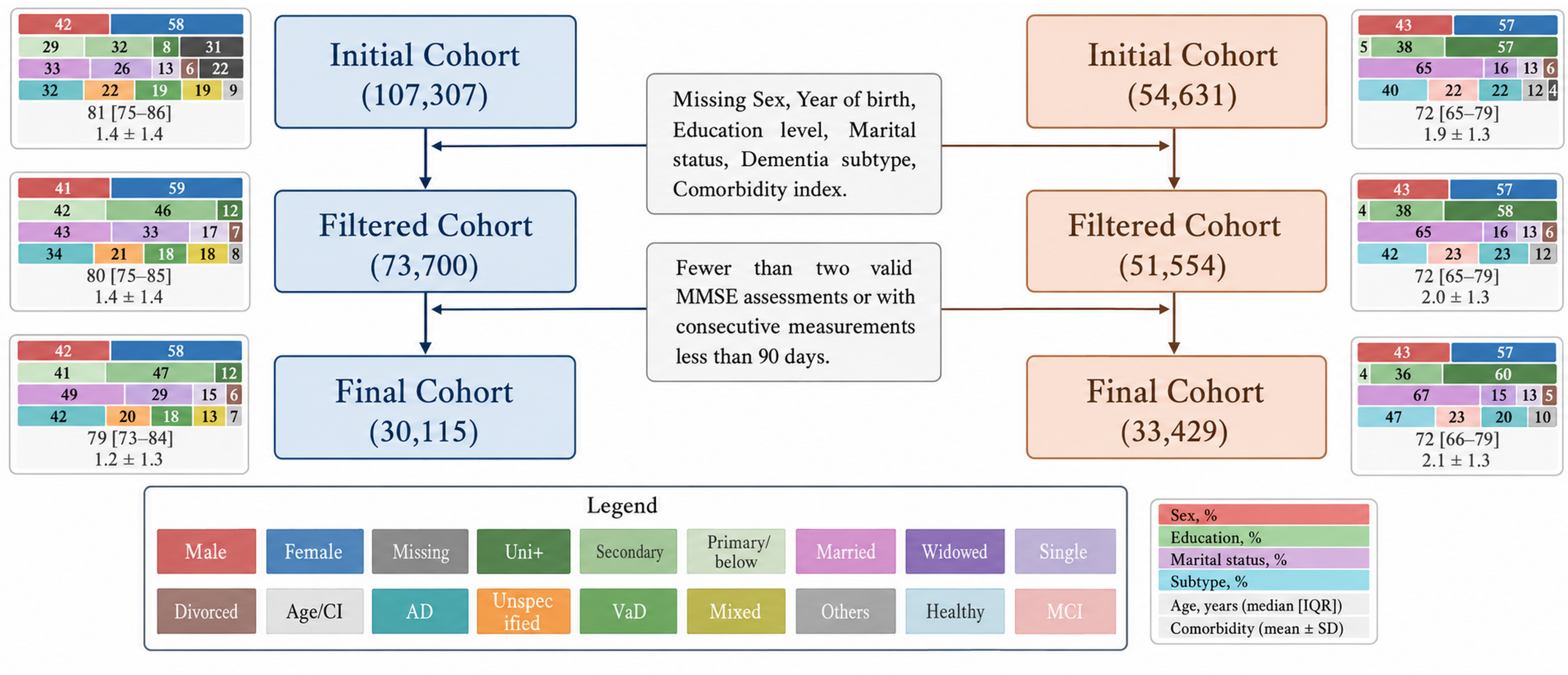

Figure S1. Participant-selection process in SveDem and NACC, including demographic and diagnostic distributions after each filtering step.

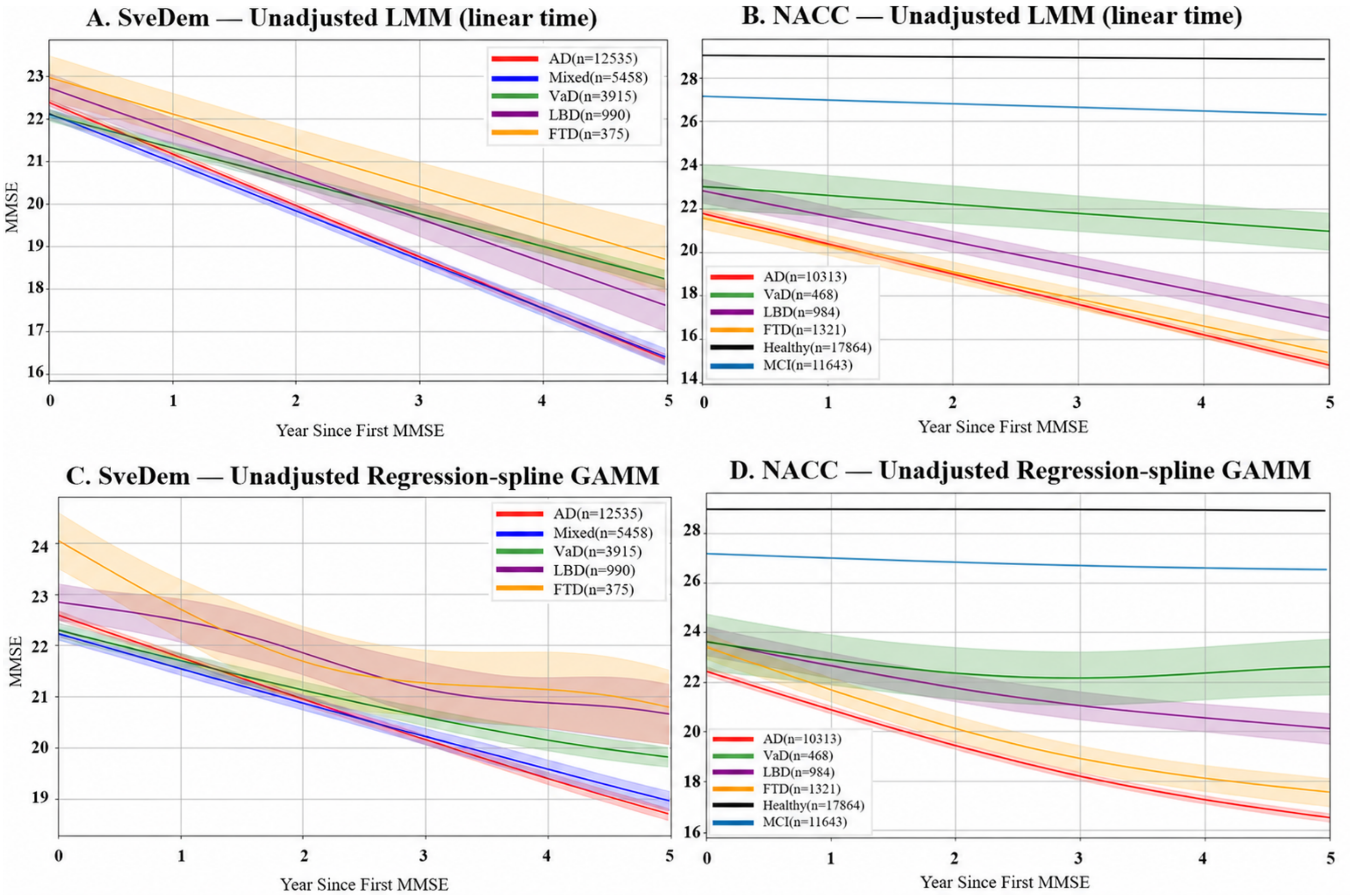

Figure S2. Subtype-specific MMSE trajectories estimated using unadjusted LMM and unadjusted regression-spline GAMM. Panels A and B show unadjusted LMM trajectories in SveDem and NACC; panels C and D show unadjusted regression-spline GAMM trajectories. Curves represent fixed-effect predictions, and shaded bands indicate 95% confidence intervals. Cognitively unimpaired participants and MCI are shown only for NACC. Numbers in the legends indicate participants recorded in each subtype at one or more retained visits. Because NACC classification was visit-specific, categories may overlap across follow-up. In the figure, “Healthy” denotes cognitively unimpaired participants.

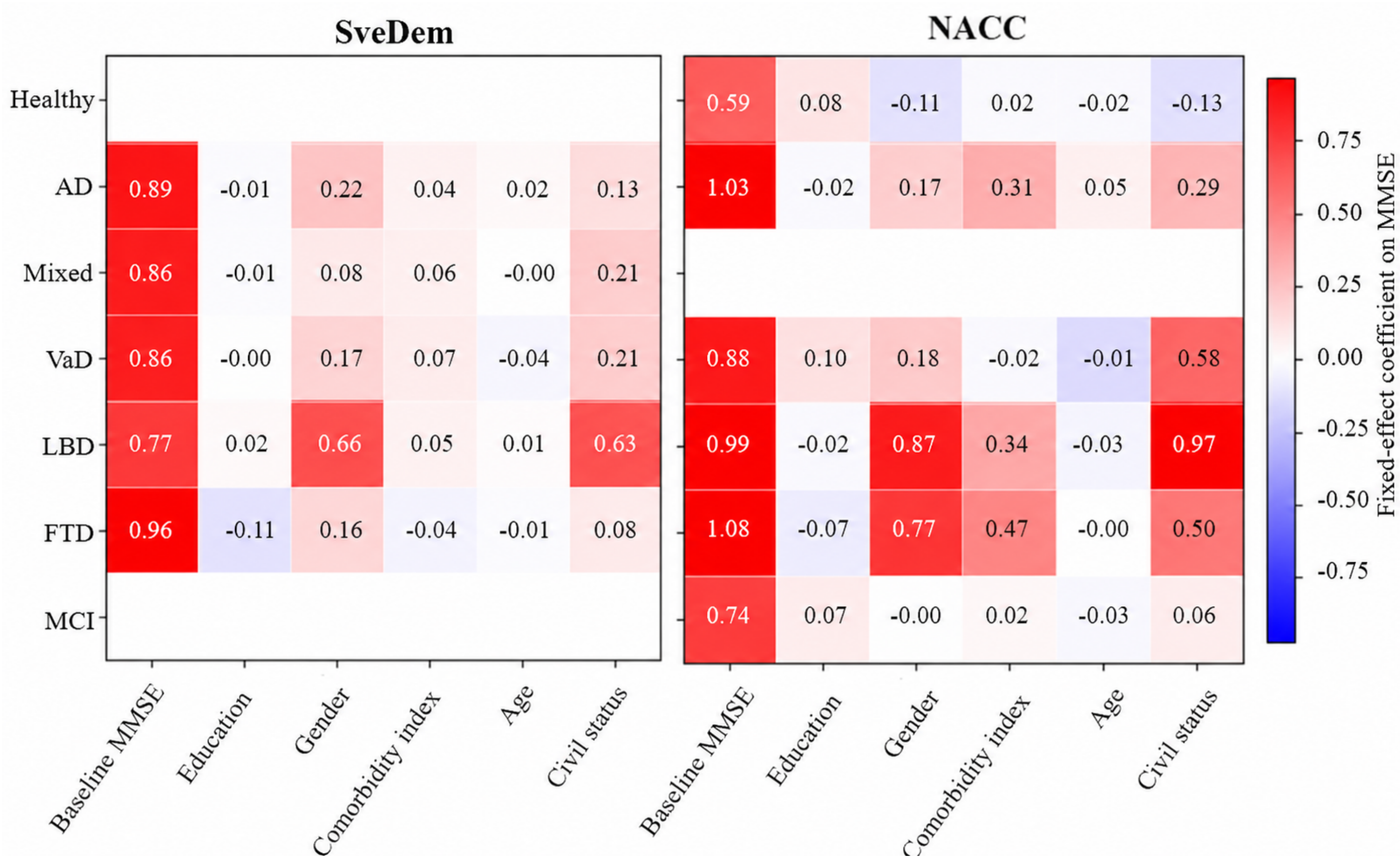

Figure S3. Fixed-effect coefficients from adjusted regression-spline GAMMs by subtype and cohort. Cells show estimated coefficients for centered baseline MMSE, education (years), sex (male vs female), comorbidity count, age at baseline, and civil status (non-married vs married/cohabiting). Coefficients are presented for descriptive purposes and should not be interpreted causally. In the figure, "Healthy" denotes cognitively unimpaired participants.
